# High-temperature infrared camouflage with efficient thermal management

**DOI:** 10.1038/s41377-020-0300-5

**Published:** 2020-04-14

**Authors:** Huanzheng Zhu, Qiang Li, Chunqi Zheng, Yu Hong, Ziquan Xu, Han Wang, Weidong Shen, Sandeep Kaur, Pintu Ghosh, Min Qiu

**Affiliations:** 1 State Key Laboratory of Modern Optical Instrumentation, College of Optical Science and Engineering, Zhejiang University, Hangzhou, 310027 China; 2 Key Laboratory of 3D Micro/Nano Fabrication and Characterization of Zhejiang Province, School of Engineering, Westlake University, 18 Shilongshan Road, Hangzhou, 310024 China; 3 Institute of Advanced Technology, Westlake Institute for Advanced Study, 18 Shilongshan Road, Hangzhou, 310024 China

**Keywords:** Nanophotonics and plasmonics, Mid-infrared photonics

## Abstract

High-temperature infrared (IR) camouflage is crucial to the effective concealment of high-temperature objects but remains a challenging issue, as the thermal radiation of an object is proportional to the fourth power of temperature (*T*^4^). Here, we experimentally demonstrate high-temperature IR camouflage with efficient thermal management. By combining a silica aerogel for thermal insulation and a Ge/ZnS multilayer wavelength-selective emitter for simultaneous radiative cooling (high emittance in the 5–8 μm non-atmospheric window) and IR camouflage (low emittance in the 8–14 μm atmospheric window), the surface temperature of an object is reduced from 873 to 410 K. The IR camouflage is demonstrated by indoor/outdoor (with/without earthshine) radiation temperatures of 310/248 K for an object at 873/623 K and a 78% reduction in with-earthshine lock-on range. This scheme may introduce opportunities for high-temperature thermal management and infrared signal processing.

## Thermal camouflage: hiding high-temperature objects

The infrared signal from high-temperature structures such as jet engines and ship funnels could be significantly concealed by a composite material that reduces the radiation temperature of internally hot objects. Such “infrared camouflaging” is especially significant for military and civil applications, to reduce the threat from heat-seeking missiles and evade thermal imaging systems. Researchers in China, led by Qiang Li at Zhejiang University, built their layered thermal camouflage from a silica aerogel and a germanium/zinc sulfide blend. The highly porous aerogel forms effective insulation. The germanium/zinc sulfide layers achieve radiative cooling by emitting energy at wavelengths outside the atmosphere’s window. In tests on objects with an internal temperature of 600 °C, the camouflage material reduced the surface temperature and the radiation temperature to 137 °C and 37 °C, respectively.

## Introduction

Infrared (IR) camouflage technology aims to conceal the IR signature of objects and render them invisible to potential threats with IR detectors, including thermal imaging systems, heat-seeking missiles, IR missile warning satellites, etc.^1–4^. According to Planck’s law, any object with a temperature above absolute zero emits thermal radiation, which is mainly located in the mid-infrared (MIR) range in most cases^5^. In particular, the thermal radiation of an object is proportional to the surface emittance (*ε*) and the fourth power of temperature (*T*). Therefore, IR camouflage for high-temperature objects (e.g., converging nozzles of aircrafts (~950 K)^6^ and funnels of naval ships (~680 K)^7^) is not only highly challenging but also urgently demanded.

IR camouflage can be achieved by controlling either the surface emittance or the surface temperature of an object. To control the surface emittance, nanostructure-based surfaces (e.g., metasurfaces^3,8^ and metallic-dielectric nanowires^9^) or films (metal^10^, semiconductor^11,12^, and multilayer films^13–17^) are demonstrated with low-surface emittance over the whole IR range, and yet the radiative heat transfer is blocked, causing severe heat instability^18^. Wavelength-selective emitters^19–25^ with radiative cooling^26–31^ in the non-atmospheric window (5–8 μm)^18,20,32^ are adopted to mitigate the heat instability without influencing the IR camouflage. However, they cannot operate at high temperature (<523 K)^18,20,32^. To control the surface temperature, thermal insulators^33^, phase-change materials^33^, and transformation thermotics^34–37^ have been proposed. Thermal insulators with low-thermal conductivity and low IR transparency can be applied to hide the IR radiation of objects, but the accompanying high absorption is contradictory to the requirement for low-surface emittance. IR camouflage with phase-change materials and transformation thermotics has been demonstrated at moderate (<573 K)^33^ and low (<373 K)^35^ temperatures, respectively. Moreover, the IR camouflage based on the phase-change material cannot be maintained after a complete phase change. Consequently, advanced technology for high-temperature IR camouflage still needs to be explored.

In this paper, we experimentally demonstrate high-temperature IR camouflage with efficient thermal management by combining wavelength-selective emitters and thermal insulators. The radiative cooling (*ε*~0.58) in the non-atmospheric window (5–8 μm) by the wavelength-selective emitter together with thermal insulation by the silica aerogel can effectively reduce the surface temperature of a high-temperature object (873 K) to 410 K. The indoor/outdoor (corresponding to with/without earthshine case) radiation temperatures are reduced to 310/248 K at object temperatures of 873/623 K, and the with-earthshine lock-on range is reduced by 76.9% compared with the case without camouflage.

## Results

### Scheme for high-temperature IR camouflage

**Fig. 1:**
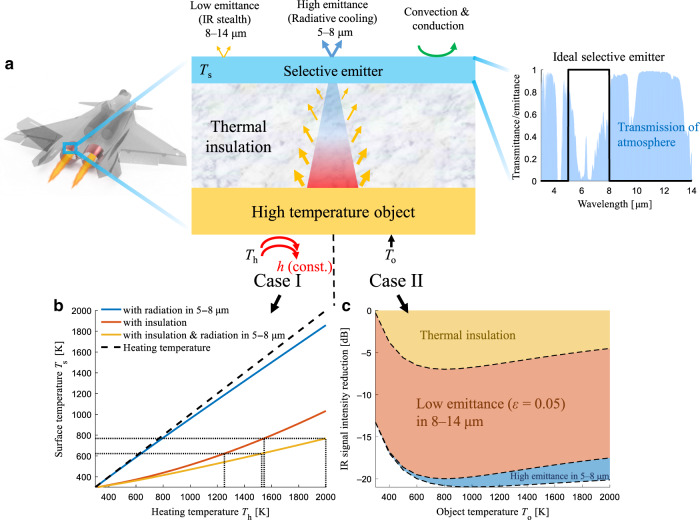
Scheme for high-temperature IR camouflage. **a** Sketch of the scheme combining a wavelength-selective emitter and a thermal insulator for high-temperature IR camouflage, with (left) a typical application on objects requiring high-temperature IR camouflage and (right) the emittance spectrum of an ideal wavelength-selective emitter along with the atmospheric transmittance spectrum. **b** Theoretical surface temperature *T*_*s*_ reduced by thermal insulation and/or radiative cooling in the 5–8 μm wavelength range for Case I in (**a**). **c** Contribution to the IR signal intensity reduction of thermal insulation (orange area), low emittance (*ε* = 0.05) in 8–14 μm (red area) and high emittance in 5–8 μm (blue area) for Case II in (**a**), at different object temperatures *T*_*o*_

The scheme for high-temperature IR camouflage, which combines a thermal insulator and a wavelength-selective emitter, is shown in Fig. 1a. The thermal insulator is directly fixed on the high-temperature object, and the selective emitter is placed on top of the thermal insulator. According to the requirement of IR opacity on the thermal insulator^38^, the thermal radiation from the high-temperature object is blocked, and the thermal radiation from the thermal insulator itself decreases with temperature. High broadband radiation covering the whole MIR range is desirable for radiative cooling of high-temperature objects^27^, while low radiation in the atmospheric window (3–5 and 8–14 μm) is required for IR camouflage. Therefore, for simultaneous IR camouflage and radiative cooling, the emittance of wavelength-selective emitters in the atmospheric window (3–5 and 8–14 μm) should be low, while that in the non-atmospheric window (5–8 μm) should be high (see the spectrum in Fig. 1a).

To demonstrate the IR camouflage effect of this scheme, two types of thermal boundary conditions (Case I and Case II) are considered in simulations with COMSOL Multiphysics (see the heat transfer simulation details in Section S1 in the Supporting information). In Case I, the lower surface of the high-temperature object is regarded as a convection surface, which is convectively heated by a variable high-temperature source *T*_*h*_ with a constant heat transfer coefficient *h* = 500 W/(m^2^ K). In Case II, the surface of the high-temperature object is a boundary with variable surface temperature *T*_*o*_. In both cases, the thermal insulation is modeled with a 1-cm-thick (experimental value) silica aerogel layer with ultralow thermal conductivity^39,40^ (0.017 W/(m K) at 400 K; see the temperature-dependent thermal conductivity of silica aerogel in Fig. S1 in the Supporting information). Under the conditions of Case I, the surface temperatures *T*_*s*_ of the object are compared for radiative cooling in the non-atmospheric window (5–8 μm), thermal insulation and their combined effect (Fig. 1b). The combination of thermal insulation and radiative cooling shows the maximum surface temperature reduction (orange line in Fig. 1b) with respect to the heating temperature. At the highest heating temperature of 2000 K, the surface temperature reductions with only radiative cooling, only thermal insulation and their combination are 142 K, 967 K, and 1232 K, respectively. As the heating temperature increases, the thermal radiation in the non-atmospheric window becomes more significant, leading to increased surface temperature reduction. When the heating temperature is higher than 800 K, the thermal radiation contributes more than 20% of the total heat flux. For only radiation in the non-atmospheric window (blue line), the surface temperature is close to the heating temperature (black dashed line), as the dissipation flux, including natural convection and thermal radiation, is small. For the same surface temperature (623 or 769 K, dotted lines in Fig. 1b), the corresponding heating temperature for the combination of radiation and insulation (1525 or 2000 K) is much higher than that for only insulation (1252 or 1543 K). Therefore, combining thermal insulation and radiation in the non-atmospheric window can significantly increase the applicable object temperature range for IR camouflage.

In Case II, the contributions of thermal insulation, low emittance in the atmospheric window (8–14 μm) and radiative cooling in the non-atmospheric window (5–8 μm) to IR camouflage are compared (Fig. 1c, and see corresponding surface temperature *T*_*s*_ in Fig. S2 in the Supporting information). The IR signal intensity of an object is estimated by integrating the IR radiant exitance in the atmospheric window (8–14 μm):

1 $$I = 10\log _{10}\left( {{\int}_{{8\, {\upmu} {\rm{m}}}}^{{14\, {\upmu} {\rm{m}}}} {\varepsilon \left( \lambda \right)M_{bb}\left( {\lambda ,T} \right)\rm{d}\lambda } } \right)$$

where *M*_*bb*_(*λ*, *T*) is the blackbody spectral radiant exitance at a given temperature *T* and *ε*(*λ*) is the surface emittance. For comparison, the IR signal intensity for the high-temperature blackbody is regarded as the base (0 dB). In the case of only thermal insulation with unity surface emittance, the surface temperature is effectively reduced, and thus, the IR signal intensity is reduced by more than 5 dB (orange area) when the temperature is higher than 500 K. Assuming the surface emittance is reduced to *ε* = 0.05 in the atmospheric window (8–14 μm), the IR signal is further reduced by 13 dB (red area). With radiative cooling in the non-atmospheric window (5–8 μm), the surface temperature further decreases, and its contribution to IR camouflage is more significant when the object temperature is high (blue area). When the object temperature is 1000 K, the IR signal intensity can be reduced by 21 dB with simultaneous thermal insulation, low emittance in the atmospheric window and radiative cooling in the non-atmospheric window, demonstrating the applicability of high-temperature camouflage via effective thermal management.

### Structure design and measurement

**Fig. 2:**
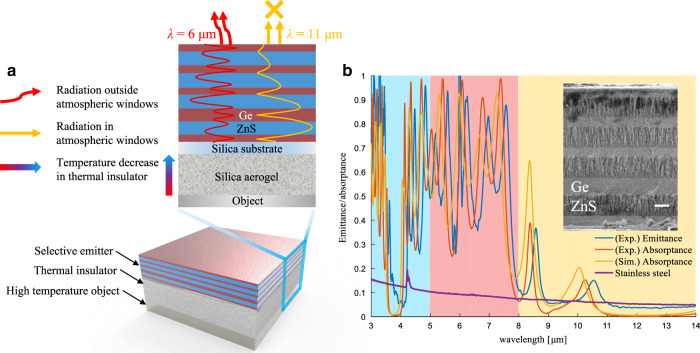
Ge/ZnS multilayer film-based wavelength-selective emitter. **a** Designed structure with a silica aerogel as the thermal insulator and Ge/ZnS multilayer films as the wavelength-selective emitter. The normalized electric field intensity is shown for a 6 μm (red curve)/11 μm (yellow curve) wavelength with high/low emittance. **b** Measured emittance/absorptance spectrum of the Ge/ZnS multilayer film-based wavelength-selective emitter compared with the simulated absorption spectrum and measured absorptance of polished stainless steel. The inset shows the SEM image of the fabricated Ge/ZnS multilayer film (nine layers), with a scale bar of 1 μm

To achieve high-temperature IR camouflage, a wavelength-selective emitter composed of alternating Ge/ZnS multilayer films is designed, and a commercially available silica aerogel blanket with high emittance (see Section S6 in the Supporting information) is chosen as the thermal insulator (Fig. 2a; see the optical and IR images in Fig. S1). The thicknesses of the Ge/ZnS (blue/red blocks in Fig. 2a) multilayer films are optimized as 0.693/1.26/0.693/1.26/0.693/1.26/1.134/1.323/0.659 μm (from top to bottom). The refractive indexes of Ge and ZnS in the MIR wavelength range are ~4 and 2.2, respectively (Fig. S3 in the Supporting information). The corresponding normalized electric field distributions at wavelengths of 6 and 11 μm are shown in Fig. 2a. For *λ* = 11 μm in the atmospheric window (orange curve), high reflectance is achieved with the distributed Bragg reflector in the upper six alternating Ge/ZnS layers, and the reflectance further increases in the bottom three layers (see reflectance spectrum in Fig. S3 in the Supporting information). The thermal radiation from the silica substrate at *λ* = 11 μm is effectively blocked, as indicated by the decaying electric field intensity (Fig. 2a). For *λ* = 6 μm in the non-atmospheric window (red curve), the transmittance of the multilayers is high, and therefore, the thermal radiation from the substrate is not blocked, contributing to the high emittance. The reflectance spectra of the wavelength-selective emitter are also not sensitive to the angle (calculated and measured reflectance spectra in Figs. S4 and S5, respectively).

The wavelength-selective emitter is experimentally fabricated by depositing alternating multilayer films of Ge and ZnS on a thin silica substrate (inset in Fig. 2b). The emittance of the wavelength-selective emitter is compared with that of polished stainless steel, which shows low-surface emittance and is extensively applied in military objectives. For the atmospheric window (8–14 μm), the band emittance (*ε*_8–14_, see band emittance in Section S3 in the Supporting information) at 573 K and the absorptance are 0.078 and 0.022, respectively, similar to the simulated absorptance of 0.094. For the non-atmospheric window (5–8 μm), the measured band emittance (*ε*_5–8_) at 573 K, the measured absorptance and the simulated absorptance are 0.580, 0.545, and 0.575, respectively. The band emittances *ε*_5–8_ and *ε*_8–14_ of stainless steel are 0.087 and 0.060, respectively. The band emittance *ε*_5–8_ of the wavelength-selective emitter is much higher than that of stainless steel without wavelength-selective radiation, while their band emittances *ε*_8–14_ are similar.

### High-temperature IR camouflage with earthshine

**Fig. 3:**
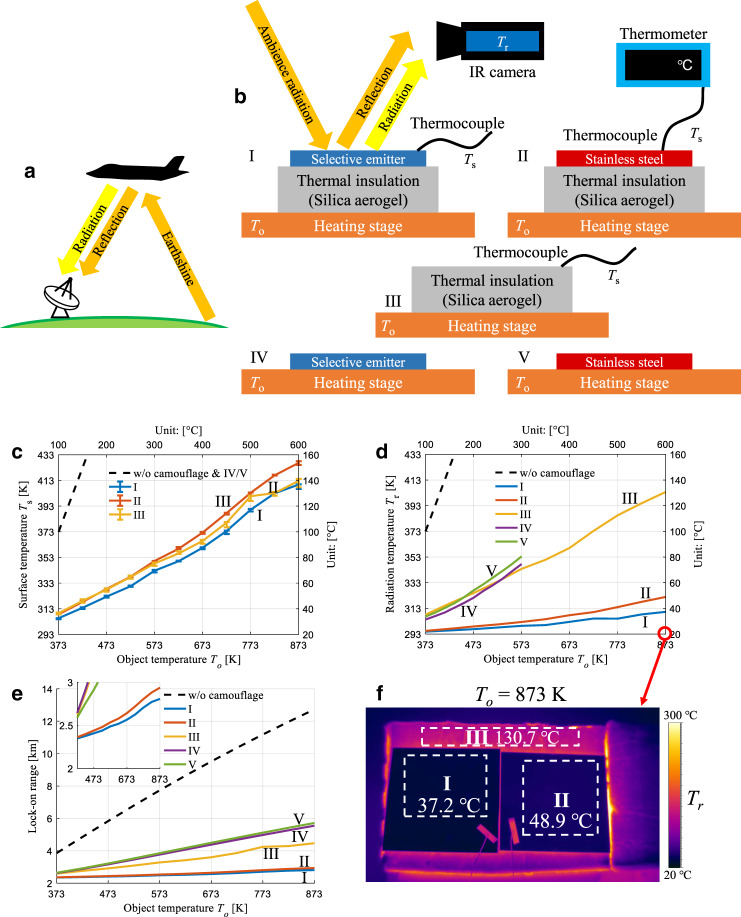
High-temperature IR camouflage with earthshine. **a** A typical situation in which the earthshine is reflected by the object. Both reflection of earthshine and radiation from the object are received by the IR detector. **b** Schematics for indoor measurement (both reflection of earthshine and radiation from the object are considered) of the surface temperature with a thermocouple and the radiation temperature with an IR camera. The measurement is conducted for five samples: I. selective emitter on silica aerogel, II. stainless steel on silica aerogel, III. bare silica aerogel, IV. selective emitter directly placed on the heating stage, and V. stainless steel directly placed on the heating stage. **c** Error bar plot of measured surface temperature *T*_*s*_ for samples I–III versus object temperature *T*_*o*_ (temperature of the heating stage) for indoor measurements. **d** Measured radiation temperature *T*_*r*_ for all samples versus object temperature *T*_*o*_ for indoor measurement. For object temperatures higher than 473 K, the surface of the stainless steel (sample V) is oxidized, and therefore, the object temperature limit for sample IV/V is only 573 K. **e** Lock-on range with earthshine calculated with measured surface temperature, measured emittance and reflected earthshine in atmospheric window for all samples, compared with situation without camouflage. **f** IR images for samples I–III at the highest object temperature of 600 °C (873 K), and average radiation temperatures are indicated in the dashed boxes

The IR detector positioned under the object collects both radiation and reflected earthshine from the object^41^, as shown in Fig. 3a. Indoor measurements are employed to imitate this situation, as both the radiation emitted from the sample and the ambient radiation reflected by the sample are collected (Fig. 3b). To demonstrate the surface temperature reduction and the IR camouflage performance for high-temperature objects, both the surface temperature and the radiation temperature of the wavelength-selective emitter on silica aerogel are measured (sample I). For comparison, polished stainless steel on silica aerogel (sample II), only silica aerogel (sample III), and selective emitter and stainless steel directly on the high-temperature object (samples IV and V) are used. The surface temperatures *T*_*s*_ of samples I–III are shown in Fig. 3c (the surface temperatures of samples IV and V are close to the object temperature *T*_*o*_ and therefore not provided). For these three samples, the surface temperatures are significantly lower than the object temperature due to the excellent thermal insulation of the silica aerogel layer. At the highest object temperature of 873 K, the surface temperature of sample I (409.8 K) is 16.9 K lower than that of sample II (426.7 K) due to radiative cooling in the non-atmospheric window for sample I.

The radiation temperature monitored by the IR camera, which indicates the collected total radiation intensity integrated in the 8–14 μm range, is employed to demonstrate the IR camouflage performance (Fig. 3d). At the highest object temperature of 873 K, the radiation temperature of sample I (310.4 K) is 11.7 K lower than that of sample II (322.1 K). The IR images of samples I and II (Fig. 3f) also show a radiation intensity reduction of sample I by approximately 15% (0.72 dB) compared with sample II in the atmospheric window (200 W/m^2^ and 236 W/m^2^ for sample I and sample II, respectively). The low-surface temperature and low band emittance (*ε*_8–14_) contribute to the lower radiation temperature of sample I. The maximum heating temperature for samples IV and V is 573 K, beyond which the samples are oxidized (Fig. S7 in Supporting information). At an object temperature of 573 K, the radiation temperature of sample I (299.6 K) is 54.1 K lower than that of sample V (353.7 K), corresponding to a 51% (3.14 dB) reduction in the band radiation intensity.

To quantitatively evaluate the IR camouflage performance, the IR lock-on range, which is an extensively employed indicator for aircraft, is assessed. The IR lock-on range is dependent on both the radiation intensity of the object and the reflected earthshine radiation^42^ (see the lock-on range calculation in Section S4 in the Supporting information). The object can only be detected when the distance between the object and the detector is within the lock-on range; a small lock-on range is therefore desirable for enhanced IR camouflage performance. The lock-on ranges considering the earthshine for all five samples are calculated with the measured surface temperature and emittance (Fig. 3e). At the highest object temperature of 873 K, the lock-on range of sample I (2.81 km) is shorter than those of sample II (2.94 km), sample V (5.72 km) and the sample without camouflage (12.73 km) by 4.5% (inset in Fig. 3e), 50.9% and 77.9%, respectively. The increase in the lock-on range with the object temperature for sample I is slower than that of the other samples and the samples without camouflage due to its lowest surface temperature and low-surface emittance. Sample I, combining a thermal insulator and a wavelength-selective emitter, shows much better high-temperature IR camouflage performance than the widely used stainless steel surface.

### High-temperature IR camouflage without earthshine

**Fig. 4:**
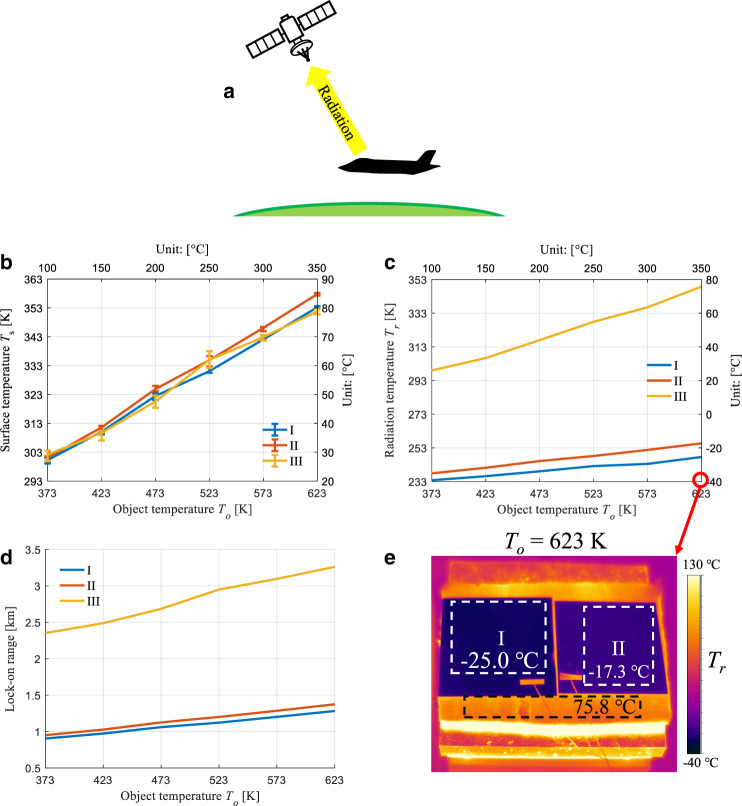
High-temperature IR camouflage without earthshine. **a** A typical situation in which the earthshine is not considered and only radiation from the object is received by the IR detector. **b** Measured surface temperature *T*_*s*_ for samples I–III (for case IV/V, the surface temperature is close to the heating temperature and thereby not considered here) in Fig. 3b versus object temperature *T*_*o*_ (temperature of heating stage) for outdoor measurement (only radiation from object itself is considered). **c** Measured radiation temperature *T*_*r*_ versus object temperature *T*_*o*_ for outdoor measurements. **d** Lock-on range without earthshine, calculated with measured surface temperature and emittance in atmospheric window. **e** IR images for samples I–III at the highest object temperature of 350 °C (623 K), and average radiation temperatures are indicated in the dashed boxes

For the IR detector positioned over the object, there is no reflected earthshine by the object, as shown in Fig. 4a. In this situation, only the radiation from the object is collected by the IR detector. The outdoor experiment on a clear night is performed to imitate this situation, and samples I–III are considered. For samples IV and V, the surface temperatures are close to the heating temperature and are therefore not considered here. At the highest object temperature of 623 K, the surface temperature of sample I is 353.2 K, which is only slightly lower than that of sample II (357.8 K), as shown in Fig. 4b. As the outdoor ambient temperature (11.9–13.1 °C) is lower than the indoor ambient temperature (~21 °C), the surface temperature in the outdoor measurement is lower than that in the indoor measurement at the same heating stage temperature (object temperature). At the highest object temperature of 623 K, the radiation temperature of sample I (248.2 K) is 7.7 K lower than that of sample II (255.9 K), corresponding to an 18% (0.86 dB) reduction in the integrated IR signal intensity, as shown in Fig. 4c. The IR image captured with the IR camera at the highest object temperature of 623 K (Fig. 4e) also indicates the lowest radiation temperature for sample I with simultaneous thermal insulation and wavelength-selective emission.

For the situation without earthshine, the lock-on ranges for samples I and II with low-surface emittance in the atmospheric window are significantly reduced (Fig. 4d). At the highest object temperature of 623 K, the lock-on range for sample I is only 1.28 km, which is approximately half that of the samples with earthshine (2.52 km). For sample III, the lock-on range is not significantly influenced by the earthshine, as its surface emittance is high. The lock-on ranges for sample III with and without earthshine at an object temperature of 623 K are 3.42 and 3.26 km, respectively. Consequently, the scheme combining thermal insulation and wavelength-selective emission presents excellent high-temperature IR camouflage performance in situations with and without earthshine. Due to the radiative cooling in the non-atmospheric window, both the indoor (with earthshine) and the outdoor (without earthshine) experiments demonstrate enhanced IR camouflage performance by combining thermal insulation and wavelength-selective emission in comparison with polished stainless steel as a common broadband low-emittance surface.

## Discussion

A scheme combining a thermal insulator and a wavelength-selective emitter with radiative cooling in the non-atmospheric window is demonstrated for high-temperature IR camouflage. First, the introduction of wavelength-selective emissive surfaces offers compatibility for IR camouflage and thermal management, as the conventional high MIR emittance requirement of thermal management is in conflict with the low MIR emittance requirement of IR camouflage. Second, the combination of thermal insulation and wavelength-selective emission provides an approach for optimized thermal management, which is of vital importance not only for high-temperature camouflage but also for other thermal management applications^43–47^. Third, wavelength-selective emitters based on multilayer films and silica aerogels for thermal insulation are easy to fabricate and thus have potential for large-area applications. Last, the temperature endurance for the combination of thermal insulation and wavelength-selective emission promises an even higher applicable object temperature than that already demonstrated (873 K). The Ge/ZnS multilayer film can stand at 623 K for 1 h (Supporting information Fig. S6); therefore, the object temperature can be well above 623 K if the silica aerogel is properly chosen (as shown by the dotted line in Fig. 1b). It has been shown that the silica aerogel has long-term temperature endurance at 923 K^48^. Ultimately, our scheme may open opportunities for further developments of energy-efficient MIR optical materials and devices^49,50^.

## Materials and methods

### Selective emitter fabrication

The Ge/ZnS multilayer film was deposited with E-beam evaporation on a silica substrate, with deposition rates of 0.5 nm/s (Ge) and 1.5 nm/s (ZnS).

### Emittance/absorptance spectrum measurements

The emittance/absorptance was measured with FTIR (Vertex 70, Brucker) with DTGS/MCT detectors.

### Radiation/surface temperature measurements

The radiation temperature was measured with an IR camera (Blackbird precision sl, Jenoptik) with a detection wavelength range of 8–14 μm. The surface temperature was measured by thermocouples (5TC-TT-K-30-36, Omega) with a thermometer (THTZ408R, Tenghui, Ningbo). The thermocouples were attached to the upper surface of the samples (I. Selective emitter on aerogel, II. Stainless steel on aerogel, III. Bare aerogel). For object temperatures higher than 473 K, the surface of stainless steel became yellow due to possible oxidization (see Supporting information Fig. S7); therefore, the object temperature limit for sample IV/V was only 573 K.

### Simulations

The heat transfer simulation was conducted with a heat transfer module and surface-to-surface radiation module in COMSOL Multiphysics. The electric field distribution was calculated with a radio frequency module (electromagnetic waves, frequency domain) in COMSOL Multiphysics.

## Supplementary information

Supporting Information
