# Supplementary material for: High-temperature infrared camouflage with efficient thermal management
Source: Light Sci Appl. 2020 Apr 14;9:60. doi: 10.1038/s41377-020-0300-5 (PMC7156722; doi:10.1038/s41377-020-0300-5)
Supplement: Supplementary file 1 — Supporting Information [file 41377_2020_300_MOESM1_ESM.docx]

**Supporting Information**

**High-temperature infrared camouflage with efficient thermal management**

Huanzheng Zhu^1^, Qiang Li^1, a)^, Chunqi Zheng^1^, Yu Hong^1^, Ziquan Xu^1^, Han Wang^1^, Weidong Shen^1^, Sandeep Kaur^1^, Pintu Ghosh^1^ and Min Qiu^2, 3^

*^1^State Key Laboratory of Modern Optical Instrumentation, College of Optical Science and Engineering, Zhejiang University, Hangzhou 310027, China.*

*^2^Key Laboratory of 3D Micro/Nano Fabrication and Characterization of Zhejiang Province, School of Engineering, Westlake University, 18 Shilongshan Road, Hangzhou 310024, China.*

*^3^Institute of Advanced Technology, Westlake Institute for Advanced Study, 18 Shilongshan Road, Hangzhou 310024, China.*

a) E-mail: qiangli@zju.edu.cn

**Section S1. Heat transfer simulation**

In Fig. 1, high temperature object is modelled in two cases (Case I for Fig. 1(b), Case II for Fig. 1(c)). For the Case I, the boundary condition is convection surface condition (assuming at position z=0):

$$-k\left.\frac{\partial T}{\partial z}\right|_{z=0}=h\left[T_{h}-T\left(z=0\right)\right]$$

where *k* is the thermal conductivity, *h* is the heat transfer coefficient, *T_h_* is the heating temperature (convection temperature). While for the Case II, the boundary condition is simply the constant surface temperature:

$$T\left(z=0\right)=T_{o}$$

The thermal conductivity of insulation layer is modelled with the thermal conductivity of silica aerogel, which is shown in Fig. S1. For the temperature outside the range (300-800 K), the thermal conductivity is acquired with extrapolation with “nearest function” in COMSOL. When only thermal insulation is applied, according to the requirement of IR stealth, the surface emittance is low among the whole spectrum. Therefore, only the radiative heat dissipation from upper surface to ambience is insufficient to form temperature gradient in thermal insulator. To overcome this problem, the natural convection boundary condition is applied on the upper surface for all three conditions (with radiative heat dissipation in non-atmospheric window, with thermal insulation and both). The natural convection is set as the external flow convection on horizontal plate with upside direction (assuming at position z=*t*, *t* is the thickness of thermal insulation layer):

$$\begin{matrix} -k\left.\frac{\partial T}{\partial z}\right|_{z=t}=h_{conv}\left[T_{amb}-T\left(z=t\right)\right] \\ h_{conv}=\frac{k}{L}0.54Ra_{L}^{1/4} \end{matrix}$$

where *L* is the characteristic length of the geometry (0.025 m for a 10×10 cm square), *T_amb_* is the ambient temperature, *Ra* is the Rayleigh number at *L*.


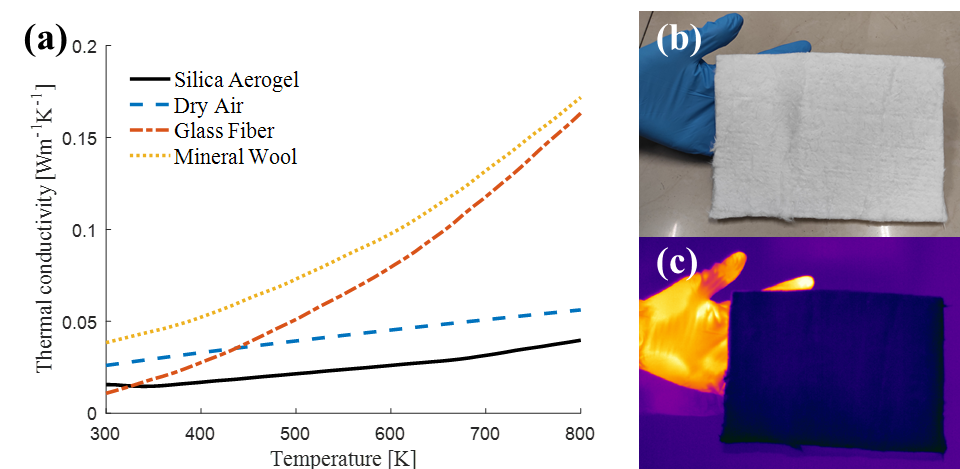


**Fig. S1**. **(a)** Thermal conductivity of silica aerogel compared with dry air^1^ and common high temperature thermal insulation materials glass fiber^2^ and mineral wool^3^. **(b)** Optical image of silica aerogel. **(c)** IR image of silica aerogel.

**Fig. S2**. Surface temperatures for different temperatures in Case II in Fig. 1(c).

**Section S2. Design of the Ge/ZnS multilayer film selective emitter**

The thickness for each layer of Ge/ZnS multilayer films is designed by the genetic algorithm, with the optimization target of maximum emittance for non-atmospheric window and minimum emittance for atmospheric window. And after optimization, some of the thickness is slightly adjusted to adjacent particular thickness of *λ*_c_/4n to form a distributed Bragg reflector (DBR), where the *λ*_c_ is the center wavelength of DBR and *n* is the refractive index. As low emittance is demanded, the *λ*_c_ is set to be 11 μm to realize broadband high reflectance in atmospheric window (8-14 μm).

The high reflectance in the atmospheric window (8-14 μm) is mainly formed with the DBR layers. From the reflectance spectra for different DBR layers (Fig. S3(a)), it can be higher reflectance can be achieved for more DBR layers. The band reflectance for 7 (upper layers), 8 (upper layers) and all 9 layers is 0.840, 0.832 and 0.906, respectively.


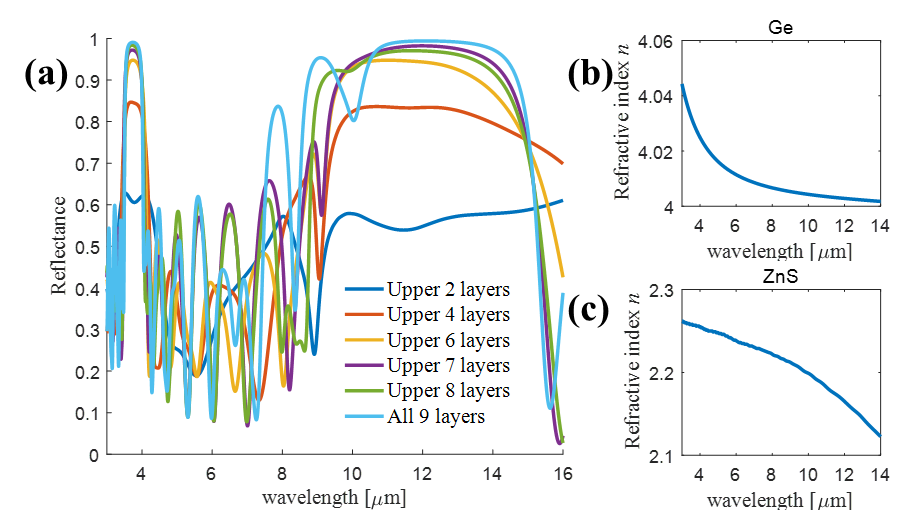


**Fig. S3**. **(a)** The reflectance spectrum of only upper 2, 4 or 6 DBR (alternating Ge/ZnS) layers, 7 layers, 8 layers and all 9 layers. **(b)** and **(c)** Refractive index for Ge and ZnS in the MIR range^4^.


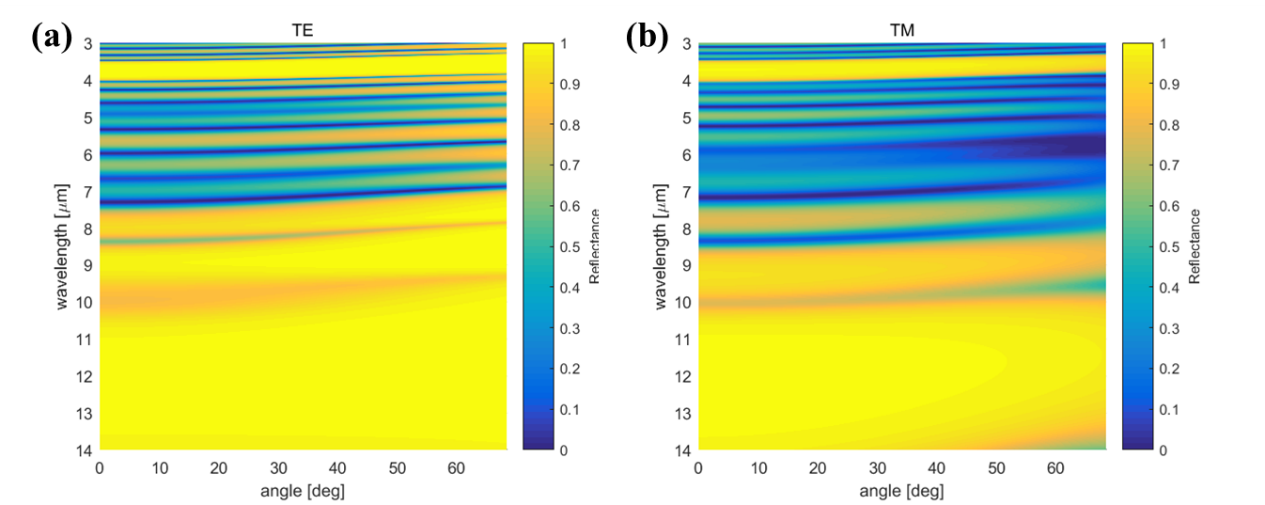


**Fig. S4**. Simulated angle dependent reflectance spectra of Ge/ZnS multilayer film selective emitter for the **(a)** TE and **(b)** TM polarization.


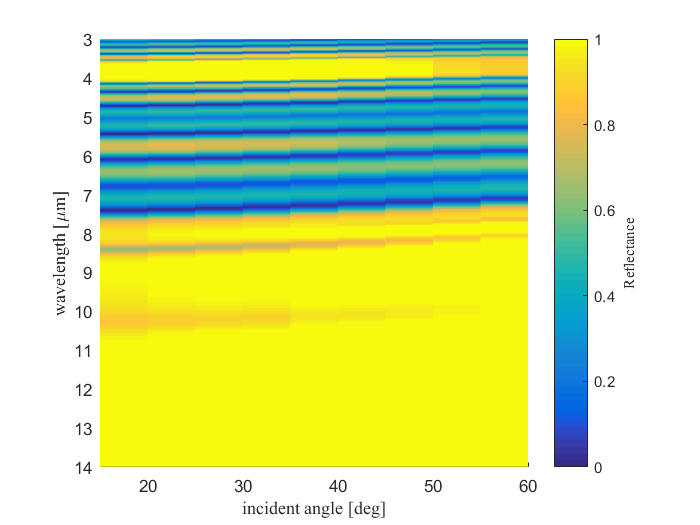


**Fig. S5**. Measured angle dependent reflectance spectra of Ge/ZnS multilayer film selective emitter.

**Section S3. Band emittance**

The band emittance of selective emitter is calculated with:

$$\varepsilon_{\lambda_{1}-\lambda_{2}}\text{=}\frac{\int_{\lambda_{1}}^{\lambda_{2}} \varepsilon\left(\lambda\right)M_{bb}\left(\lambda,T\right)\text{d}\lambda}{\int_{\lambda_{1}}^{\lambda_{2}} M_{bb}\left(\lambda,T\right)\text{d}\lambda}$$

When the surface temperature is decreased to less than 373 K, the absolute radiation intensity in mid-wave IR range is relatively lower than 8-14 μm (~51 W∙m^-2^ for 3-5 μm, ~429 W∙m^-2^ for 8-14 μm for blackbody emitter at 373 K). Therefore the emittance for the mid-wavelength IR band cannot significantly influence the IR stealth performance in this scheme.

**Section S4. Lock-on range calculation**

The lock-on range is determined by the sensitivity of the IR detector and the irradiance on the IR detector, which is dependent on the radiation intensity of object and the reflected earthshine radiation^5^:

$$\begin{matrix} R_{LO,8-14}=\sqrt{A_{obj}H_{8-14}/\left(\Omega_{obj}\cdot NEI\cdot\xi_{\min}\right)} \\ H_{8-14}=\Omega_{obj}\cdot\int_{8\mu m}^{14\mu m} \varepsilon\left(\lambda\right)M_{bb}\left(\lambda,T_{obj}\right)\tau_{atm}\left(\lambda\right)+\left[1-\varepsilon\left(\lambda\right)\right]M_{bb}\left(\lambda,T_{ear}\right)\tau_{atm}\left(\lambda\right)d\lambda\end{matrix}$$

where *H*_8-14_ is the band integrated irradiance on the IR detector calculated with the object’s surface emittance *ε*(*λ*), blackbody spectral radiant exitance at object’s surface temperature *T_obj_* and earth temperature *T_ear_*, the transmission spectrum of atmosphere *τ_atm_*(*λ*), and the effective solid angle of object *Ω_obj_*. The lock-on range *R_LO_*_, 8-14_ is then acquired with the minimum threshold value (*NEI*∙*ξ_min_*) of the IR detector, effective area of object *A_obj_*, the background radiation intensity *I_bg_* and the irradiance *H*_8-14_.

**Section S5. Temperature endurance**

**Fig. S6**. The emittance spectrum of the Ge/ZnS multilayer film before heated (blue dashed line) and after heated at 623 K for 1 hour (orange dotted line).


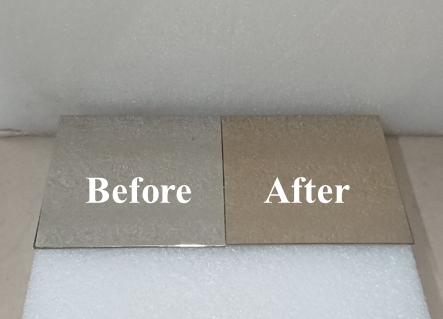


**Fig. S7**. The photos of polished stainless steel plates before and after oxidization (heated at 573 K for 30 mins).

**Section S6. Emittance of Silica Aerogel**

The emittance of silica aerogel is measured to prove that the absorptance is high and therefore transmission is low.

**Fig. S8**. Emittance spectrum of silica aerogel. The emittance is higher than 1 due to possible nonuniform temperature distribution on rough surface of silica aerogel blanket (the reference blackbody is a relatively smooth surface).

1. Engineering ToolBox. Dry Air Properties. (2005). Available at: https://www.engineeringtoolbox.com/dry-air-properties-d_973.html.

2. Warm, I. T. O. & Insulation, F. Fiberglass insulation. 1–7 (2012). Available at: https://www.engineeringtoolbox.com/fiberglas-insulation-k-values-d_1172.html.

3. Kowatsch, S. Mineral wool insulation binders. *Phenolic Resins: A Century of Progress* 209–242 (2010). doi:10.1007/978-3-642-04714-5_10

4. Palik, E. D. *Handbook of optical constants of solids (Vol.1)*. (1998).

5. Rao, G. A. & Mahulikar, S. P. Aircraft powerplant and plume infrared signature modelling and analysis. *43rd AIAA Aerosp. Sci. Meet. Exhib. - Meet. Pap.* **91**, 1397–1407 (2005).
